# Maternal antimicrobial use at delivery has a stronger impact than mode of delivery on bifidobacterial colonization in infants: a pilot study

**DOI:** 10.1038/s41372-018-0172-1

**Published:** 2018-07-24

**Authors:** Naruaki Imoto, Hiroto Morita, Fumitaka Amanuma, Hidekazu Maruyama, Shin Watanabe, Naoyuki Hashiguchi

**Affiliations:** 1 Department of Emergency and Disaster Medicine, School of Medical Science, Juntendo University, Bunkyo Ward, Tokyo, Japan; 2 Core Technology Laboratories, Asahi Group Holdings, Ltd., Sagamihara, Kanagawa Japan; 3 Department of Pediatrics, Department of Neonatology, Iwate Prefectural Iwai Hospital, Ichinoseki, Iwate Japan

## Abstract

**Objective:**

To investigate factors related to bifidobacterial colonization in early infancy, with a focus on maternal antimicrobial use at delivery.

**Study design:**

A cross-sectional pilot study was performed. Feces samples of 33 Japanese healthy infants were collected over 10 months and analyzed by next-generation sequencing to examine the diversity and abundance of the gut microbiota.

**Results:**

The beta diversity index of the gut microbiota differed significantly based on maternal antimicrobial use at delivery (*P* < 0.05). The most dominant genus was bifidobacteria, and the relative abundance of bifidobacteria in infants exposed to maternal antibiotics was significantly lower than in those who were not exposed (*P* < 0.05). In contrast, the delivery mode showed no significant relationship with gut microbiota diversity.

**Conclusions:**

Maternal antimicrobial use at delivery has a stronger effect than delivery mode on the gut microbiota, especially for colonization of bifidobacteria.

## Introduction

There are 100 trillion to 1 quadrillion bacteria consisting of 1000 bacterial species inhabiting the human intestine, and mutual metabolic activity between enterobacteria plays important roles in host health and disease onset [1–3]. Until recently, many enterobacteria could not be cultured and this has prevented detailed analyses. However, next-generation sequencing of 16S rRNA genes allows comprehensive analysis of the gut microbiota [4, 5] and has shown racial and regional differences in the microbiota composition. For example, Japanese school children and young adults have been found to have more bifidobacteria than persons of similar age in other countries [6, 7]. However, it is unknown if dietary constituents are related to the diversity of the gut microbiota [7].

The period from birth to weaning is important for establishment of adaptive immunity and immune tolerance. The change in the composition of gut microbiota in early infancy over the first 6 months is thought to be crucial in establishing the immune system against allergy or infections, and dysbiosis during this period can lead to future development of diseases [8]. The role of bifidobacteria seems to be particularly significant. Several studies have evaluated the relationship of bifidobacterial colonization with allergic diseases, including atopic dermatitis and asthma [9–11], and bifidobacteria play a protective role in building the immune system in the intestinal mucosa [12–14]. However, the proportion of bifidobacteria in the intestine of infants in the early stage has varied among studies [15, 16].

Several factors seem to affect bifidobacterial colonization in early infancy. Delivery and nutrient intake may influence this process and affect growth in infants, based on findings of decreased bifidobacteria in infants delivered by Cesarean section and increased bifidobacteria in breastfed infants [17–20]. However, other studies have shown no effects of these factors [5, 21]. Intravenous antimicrobial agents are generally administered prophylactically to mothers before Cesarean delivery [22], but the effect of these agents has not been widely addressed in most previous studies on bifidobacteria colonization. Some studies have examined the effects of maternal administration of antibiotics at delivery on oral microbiota [23] and gut microbiota [24] in infants; however, the impact of maternal antimicrobial use just before delivery, including Cesarean section, on bifidobacterial colonization in early infancy remains unclear.

The objectives of this study were to analyze the gut microbiota in Japanese infants using next-generation sequencing, determine the abundance of bifidobacteria in the infants, and identify factors related to infant bifidobacterial colonization, with a focus on the impact of maternal antimicrobial treatment on the colonization. The study is based on the hypothesis that maternal antimicrobial use just before delivery, including Cesarean section, has a stronger impact than the difference in delivery mode on the gut, and especially on colonization of bifidobacteria, in early infancy.

## Methods

### Study design

This study was conducted as a pilot study prior to follow-up of the gut microbiota in infants and their mothers over a long period. A cross-sectional study was designed for analysis of enterobacteria in feces of subjects using next-generation sequencing. The subjects were 33 healthy infants who underwent a check-up 1 month after birth in Iwate Prefectural Iwai Hospital for 10 months from January to October 2016. All the infants were found to be healthy in the health check. All parents agreed to participation of their infant in the study. A sample size of approximately 30 subjects was determined to be sufficient for diversity analysis based on a preceding study [25, 26].

Several days after registration, containers for collection of feces samples were delivered by mail to their home by the Department of Emergency and Disaster Medicine, Juntendo University (Bunkyo-ku, Tokyo, Japan). Each fecal sample was collected by the subject’s parents in a test tube (Techno Suruga Laboratory, Shizuoka, Japan) containing 100 mM Tris-HCl (pH 9), 40 mM EDTA, 4 M guanidine thiocyanate, and 0.001% bromothymol, and mixed well [27]. Mixed fecal samples were delivered to a laboratory of Asahi Group Holdings (Sagamihara, Kanagawa, Japan) and stored at −80 °C until processing for DNA extraction.

### DNA extraction

For DNA extraction, the samples (2 ml) were transfer to plastic tubes and centrifuged at 14 ,000×*g* for 3 min, and then washed in 1.0 ml phosphate-buffered saline and centrifuged at 14 ,000×*g*. Pellets were resuspended in 500 μl extraction buffer (166 mM Tris/HCl, 66 mM EDTA, 8.3% sodium dodecyl sulfate, pH 9.0) and 500 μl TE buffer-saturated phenol. Three hundred milligram of glass beads (0.1 mm diameter) was added to the suspension and the mixture was vortexed vigorously for 60 s using a Multi Beads Shocker® (Yasui Kikai Corporation, Osaka, Japan). After centrifugation at 14 ,000×*g* for 5 min, 400 μl of the supernatant were purified by Maxwell® Instrument (Promega KK, Tokyo, Japan).

### Sequencing and data processing

Sequencing of the gene encoding 16S rRNA was performed with MiSeq V2 kit according to manufactured procedure (http://support.illumina.com/documents/documentation/chemistry_documentation/16s/16s-metagenomic-library-prep-guide-15044223-b.pdf). Briefly, the V4 region of the bacterial 16S rDNA was amplified by PCR with forward and reverse primer (5′-TCGTCGGCAGCGTCAGATGTGTATAAGAGACAG GTGCCAGCMGCCGCGGTAA-3′ and 5′-GTCTCGTGGGCTCGGAGATGTGTATAAGAGACAG GACTACHVGGGTATCTAATCC-3′, respectively), 5 ng of the DNA from fecal sample, and the TaKaRa Ex Taq HS Kit (TaKaRa Bio, Shiga, Japan). After the PCR products were purified by Agencourt AMPure XP (Beckman Coulter, Inc., CA, USA), the products were amplified using the Nextera Index Kit (Illumina, CA, USA). After the 2^nd^ PCR, amplified products were purified using Agencourt AMPure XP. Library was quantified, normalized and pooled in equimolar amounts according to the manufacturer’s recommendations. Sequencing was conducted using a paired-end to 2 × 150-bp cycle run on an Illumina MiSeq system and MiSeq Reagent Kit version 2 (300 Cycle).

### 16S rDNA-based taxonomic and diversity analysis

QIIME (Quantitative Insights into Microbial Ecology, http://qiime.org/) v.1.8.0. was used for filtering and analysis of sequences [28]. Quality filtering was performed using the provided fastq files and sequences with a quality score < 29 were removed. Chimeric sequences were removed using USEARCH. Assignment to operational taxonomic units (OTUs) was carried out using open-reference OTU picking with a 97% threshold for pairwise identity. After OTUs containing < 5 sequences were removed, the OTUs were classified taxonomically using the Greengenes reference database (http://greengenes.secondgenome.com/downloads/database/13_5). The total number of sequence reads retained for analysis was 4,831,105. The mean, minimum, and maximum of the reads per sample were 146,397, 76,617, and 251,215, respectively. Alpha diversity (Chao1, number of observed species, phylogenetic distance whole tree, and Shannon diversity index) within two groups and the distances between subjects (unweighted UniFrac distance as beta diversity) were also estimated using QIIME with rarefied data at 50,000 reads per sample. Beta diversity was visualized by principal coordinate analysis (PCoA).

### Data collection

The following items for infants were collected from medical records at Iwate Prefectural Iwai Hospital: gender, body weight after birth, increase in body weight after birth to medical check-up, perinatal history, delivery method, use of antibacterial agents after birth; and from a written questionnaire completed by mothers: age (days) at sample collection and nutrient intake. Items for mothers were similarly collected from medical records: age, delivery history, history of allergies (food allergy, bronchial asthma, atopic dermatitis, and allergic rhinitis), abnormal findings at delivery (including premature rupture of membrane (PROM) and group B streptococcus (GBS)-positive status), and systemic antibacterial agents taken at delivery; and from the questionnaire: other children and history of allergies (food allergy, bronchial asthma, atopic dermatitis, and allergic rhinitis). The survey and analysis were conducted from January to December 2016.

### Statistical analysis

A two-sample *t*-test using Monte Carlo permutations within QIIME was used to compare alpha diversities between groups of subjects. The significance of a difference between two groups was evaluated using a non-parametric ANOSIM (analysis of similarities) test based on unweighted UniFrac distances within QIIME. The number of permutations for both tests was set at 999 to calculate *P*-values. Spearman rank correlation analysis was used to evaluate the correlation between two continuous variables. Between-group comparison of relative abundance was performed by Mann–Whitney *U*-test. The threshold for significance was *P* < 0.05.

### Ethical standards

The authors assert that all procedures contributing to this work comply with the ethical standards of the relevant national guidelines of the Japanese government on human experimentation and with the Helsinki Declaration of 1975, as revised in 2008, and has been approved by the institutional review board of Iwate Prefectural Iwai Hospital (No. 453) and Juntendo University (No. 2015112). Informed written consent was obtained from all of the mothers. The first and last authors take complete responsibility for the integrity of the data and the accuracy of the data analysis.

## Results

Clinical characteristics of the mothers and infants are shown in Table 1. The median age of infants on the day of sample collection was 44 days (interquartile range: 41–53 days). No premature infant was included in the study. Three infants received oxygen after birth due to slight respiratory disorder. They improved after several days and were discharged in a healthy condition. All infants were checked-up 1 month after birth. None received antimicrobial agents after birth to the day of sample collection. Antimicrobial agents were given systemically to 19 mothers at delivery for Cesarean section (*n* = 9), GBS-positive status (*n* = 4), and PROM (*n* = 6). A single dose of cefazolin (1 g) was given preoperatively for all Cesarean sections just before the operation. In the GBS-positive and PROM cases, ampicillin (2 g) was given at least 4 h before delivery, followed by every 6 h until delivery. In all cases, antibiotics were administered at the dose and time defined in the clinical protocol determined by the hospital board.

**Table 1:** Clinical characteristics of infants and mothers

Characteristics	Values
Infants	33
Male infants	19 (58)
Gestational age at birth (days)^a^	275 ± 7.8
Birth weight (g)^a^	3016 ± 350
Age of infants when feces were collected (days)^b^	44 (41–52)
Cesarean section	9 (27)
Exclusively breastfed babies	22 (67)
Mixed-fed babies	10 (30)
Exclusively formula-fed babies	1 (3)
Infants with older siblings	17 (51)
Age of mothers^b^	31 (29–35)
Maternal antimicrobial use at delivery	19 (58)
Mothers with history of allergy	16 (48)

Data are shown as *n* (%) unless otherwise indicated

^a^Mean ± SD

^b^Median (interquartile range)

**Fig. 1:**
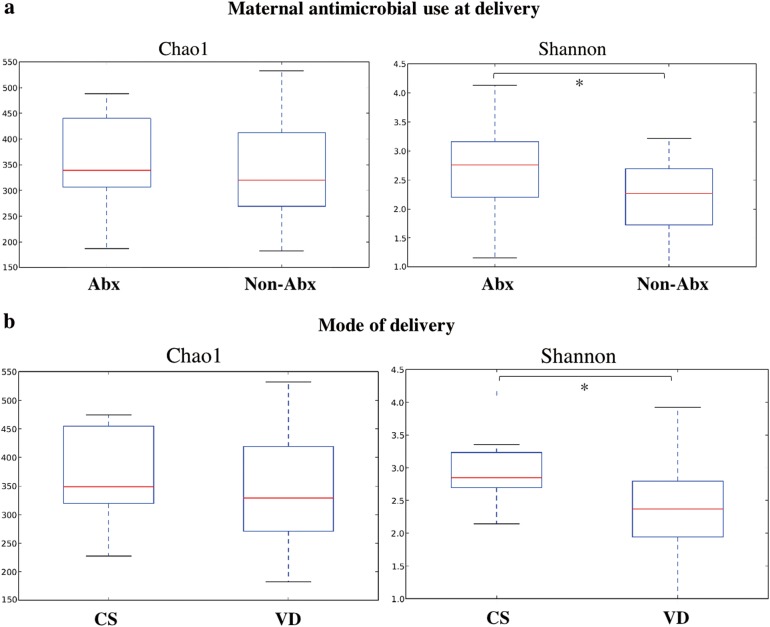
Alpha diversity index. The alpha diversity index is an indicator of diversity in race in a certain environment. The Chao1 index shows a simple comparison of numbers and the Shannon index is an indicator considering the homogeneity. **a** Comparison of infants with (*n* = 19, Abx) and without (*n* = 14, non-Abx) use of maternal antimicrobial agents at delivery. Chao1 index (*P* = 0.66), Shannon index (*P* = 0.049). Maternal antimicrobial use at delivery includes exposure to antibiotics just before Cesarean section or vaginal delivery. **b** Comparison with mode of delivery. Chao1 index (*P* = 0.53), Shannon index (*P* = 0.035) for Cesarean section (*n* = 9, CS) vs. vaginal delivery (*n* = 24, VD). *P < 0.05

**Fig. 2:**
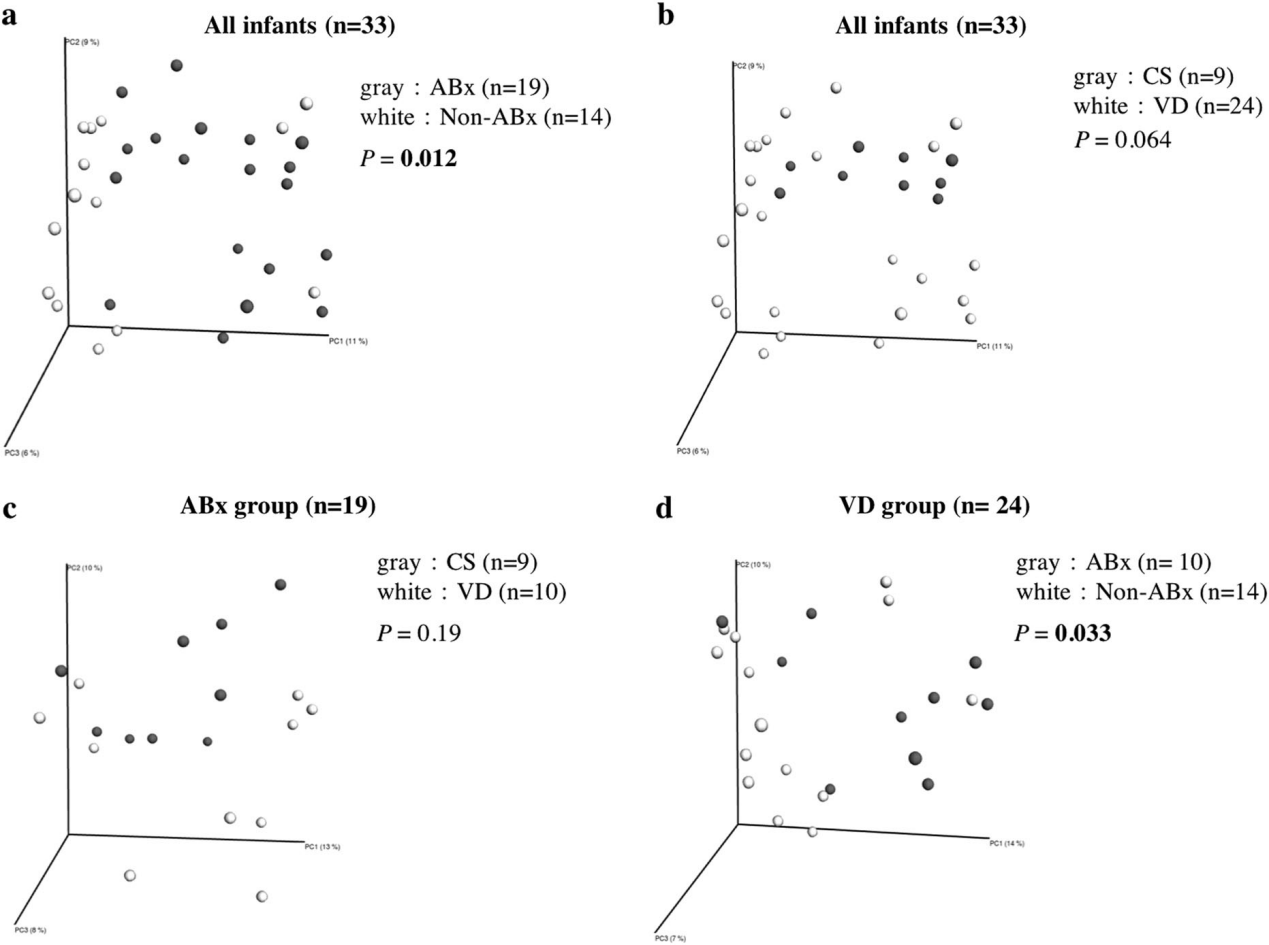
Beta diversity of the gut microbiota at the genus level. The beta diversity index is an indicator of differences in race between environments. **a** Principal coordinates analysis (PCoA) showed a significant difference between infants with (*n* = 19, Abx) and without (*n* = 14, non-Abx) use of maternal antimicrobial agents at delivery. **b** PCoA showed no significant difference for mode of delivery comparing Cesarean section (*n* = 9, CS) with vaginal delivery (*n* = 24, VD). **c** In the ABx group, there was no significant difference due to delivery mode or type of antibiotics (CS group treated with cefazolin and VD group treated with ampicillin). **d** In the VD group, there was a significant difference between the groups with and without antibiotic treatment. The threshold for significance was *P* < 0.05. Significant *P* values are shown in bold. The UniFrac distance was used for beta diversity

In feces samples of the 33 infants, the Shannon diversity index was significantly related to antimicrobial use at delivery and mode of delivery (Fig. 1). The beta diversity index was also significantly related to antimicrobial use at delivery, and in a subgroup analysis of vaginal delivery cases only, but was not significantly associated with mode of delivery (Fig. 2).

**Fig. 3:**
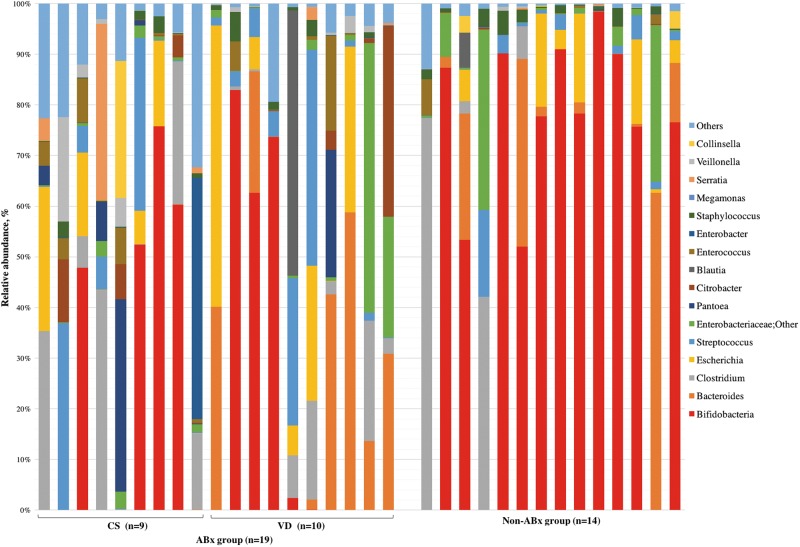
Representation of bacterial family relative abundance in the gut microbiota in 33 healthy infants (mean age 44 days). Each vertical bar represents an infant, segregated into two groups according to antimicrobial use at delivery. The ABx and Non-ABx groups include infants whose mothers did and did not receive antimicrobials at delivery, respectively. In the ABx group, CS indicates Cesarean section and VD indicates vaginal delivery. Bars are shown in the order of registration. The top 20 bacteria strains are shown and other strains are included in “others”. Bifidobacteria (indicated in red) were dominant in most infants

Among the 33 infants, the dominant bacterial genus was bifidobacteria (mean: 40.8%, standard error: ±6.8%), followed by *Bacteroides* (10.8 ± 3.2%) and *Clostridium* (9.5 ± 3.0%) (Fig. 3). A comparison of groups with and without antibiotic treatment and between delivery modes showed that the bifidobacterial abundance in infants was significantly lower in those with maternal antibiotic treatment during delivery. In a stratified analysis, there was no significant difference in bifidobacterial abundance between the Cesarean section group treated with cefazolin (*n* = 9) and the vaginal delivery group treated with ampicillin (*n* = 10). In the vaginal delivery group, there was a significant difference between infants with mothers that did and did not receive antibiotics. In contrast, the results for *Bacteroides* were opposite to those for Bifidobacterium: a significant difference in abundance was found between delivery modes, but there was no significant difference between infants with and without maternal antibiotic treatment (Table 2).

**Table 2:** Relative abundance of the six most common bacterial genera in all infants (*n* = 33) and in those with and without use of maternal antibiotics and with Cesarean or vaginal delivery

Bacterial genus	All infants (33)						ABx group (19)			VD group (24)		
	CS (9)	VD (24)	*P*	ABx (19)	Non-Abx (14)	*P*	CS (9)	VD (10)	*P*	ABx (10)	Non-ABx (14)	*P*
*Bifidobacteriaceae; Bifidobacterium*	0.06 (0.02–52.4)	58.0 (0.04–79.5)	0.13	0.06 (0.02–56.3)	77.1 (52.3–89.3)	**0.01**	0.06 (0.02–52.4)	0.06 (0.03–47.6)	0.87	0.06 (0.03–47.6)	77.1 (52.3–89.3)	**0.03**
*Bacteroidaceae; Bacteroides*	0.02 (0.01–0.04)	2.0 (0.4–26.5)	**0.01**	0.04 (0.02–18.8)	1.2 (0.04–9.3)	0.49	0.02 (0.01–0.04)	18.8 (0.5–37.8)	**0.03**	18.8 (0.5–37.8)	1.2 (0.04–9.3)	0.32
*Clostridiaceae; Clostridium*	6.2 (0.002–28.2)	0.03 (0.0–3.9)	0.18	2.6 (0.001–17.3)	0.004 (0.0–1.8)	0.16	6.2 (0.002–28.2)	1.6 (0.1–7.1)	0.46	1.6 (0.1–7.1)	0.004 (0.0–1.8)	0.29
*Enterobacteriaceae; Escherichia*	0.1 (0.07–16.5)	0.4 (0.003–9.0)	0.59	0.1 (0.003–16.7)	0.4 (0.01–5.8)	0.57	0.1 (0.07–16.5)	3.0 (0.0–21.6)	0.80	3.0 (0.0–21.6)	0.4 (0.01–5.8)	0.77
*Streptococcaceae; Streptococcus*	0.2 (0.2–6.5)	1.5 (0.1–3.9)	0.76	1.5 (0.2–6.1)	1.1 (0.07–2.8)	0.14	0.2 (0.2–6.5)	2.3 (1.2–5.5)	0.81	2.3 (1.2–5.5)	1.1 (0.07–2.8)	0.14
*Enterobacteriaceae; Other*	0.7 (0.6–2.4)	0.6 (0.2–2.4)	0.62	0.7 (0.4–2.2)	0.4 (0.2–3.1)	0.54	0.7 (0.6–2.4)	0.9 (0.3–1.8)	0.81	0.9 (0.3–1.8)	0.4 (0.2–3.1)	0.63

The relative abundance by each bacterial genus is shown as the median percentage. The interquartile range is shown in parentheses below the median percentage. VD, vaginal delivery (*n* = 9 infants); CS, Cesarean section (*n* = 24); ABx (*n* = 19) and non-ABx (*n* = 14), infants exposed and not exposed to antibiotics just before CS or VD. Significant *P* values are shown in bold. Comparison of relative abundance was tested by Mann–Whitney *U*-test

In an analysis of the association of bifidobacterial abundance with background factors of infants and mothers, the abundance was significantly lower in those without older siblings (*n* = 16) compared to those with older siblings (*n* = 17) (*P* < 0.05). There were no significant differences for exclusively breastfed infants, sex, maternal history of allergy, gestational age at birth, birth weight, age of infants when feces were collected, and age of mothers (see Supplemental Information).

## Discussion

This study suggests an impact of maternal antimicrobial use at delivery on the early colonization of bifidobacteria in the gut of infants and on the composition of the gut microbiota. The same results were found in a subgroup analysis of vaginal delivery cases. In contrast, different types of antibiotics had no impact on the abundance of bifidobacteria or on the composition of the gut microbiota. A previous study showed no effect of administration of antimicrobial agents on the gut microbiota in infants; however, antimicrobials used during late pregnancy (last month) were included in this analysis [19]. A few studies have examined the impact of intrapartum antibiotic prophylaxis in GBS-positive mothers on the gut microbiota in their infants [25, 26, 29], but to our knowledge, the relationship of the proportion of bifidobacteria in the gut of healthy infants with antimicrobial use in GBS-positive mothers and in Cesarean section and PROM cases at delivery only has not been examined previously. Antimicrobial agents administered immediately before delivery may disturb the gut microbiota in infants due to transfer to the fetus through the umbilical cord.

In the current study, the mode of delivery had less impact on bifidobacterial colonization and the composition of the gut in early infancy, compared to antimicrobial use at delivery. Some studies [5, 21] have also found no relationship between fewer bifidobacteria in infants and delivery by Cesarean section, while an effect of the delivery method on the proportion of bifidobacteria has been found in other studies [19, 20, 30]. Infants delivered by Cesarean section are not exposed to maternal bacteria because they do not transit through the maternal birth canal, and this could explain the lower level of bifidobacteria in these infants; however, these studies did not consider the effect of antimicrobial use at the time of delivery, as shown in the current study. The absence of a significant difference in the influence of Cesarean delivery on bifidobacteria in this study may have been due to the inclusion of mothers treated with antibiotics at delivery who gave natural birth. Given that intravenous antimicrobial agents are generally administered prophylactically to mothers before Cesarean delivery [22], previous studies of the effect of Cesarean delivery on bifidobacteria may have actually evaluated the influence of antimicrobial use at delivery.

There was a difference in Shannon diversity index between delivery modes, and abundance by Bacteroides was significantly lower after Cesarean section in the current study. Thus, other factors may have an influence on the gut microbiota in early infancy. To our knowledge, factors influencing abundance by Bacteroides in early infancy have not been described, and the findings of our study are interesting in this respect. Two types of beta-lactam antibiotics, cefazolin and ampicillin, were used and the antibacterial spectrum differs slightly between these drugs. Therefore, sensitivity of each bacterium to these antibiotics may be important. This study was performed to investigate bifidobacterial abundance, but it also shows the need for a study with a sufficient number of samples to deepen understanding of effects on *Bacteroides* and other bacterial genera, including their clinical significance.

Our results also suggest that the presence of older siblings may have an impact on bifidobacterial colonization in infants. The higher level of bifidobacterium in infants with older siblings is probably a reflection of higher rates of exposure to bacteria in infants with siblings compared to firstborn infants. Other external factors may also influence the gut microbiota in infants, but there is currently little evidence for these factors. Further large-scale studies are needed to examine factors determining the proportion of bifidobacteria in early infancy. This is important because a lower proportion of bifidobacteria in early infancy may be linked to future occurrence of allergic diseases [10, 12]. Several studies have shown an effect of administration of bifidobacteria in low-birth-weight infants [31, 32]. Intervention strategies, including administration of bifidobacteria in healthy infants with a lower proportion of bifidobacteria, may be needed as the next step in our investigation of the clinical importance of early colonization of bifidobacteria in infants.

The limitations of this study are as follows. The subjects lived in only one region in Japan and the results are not always consistent with other findings in Japanese subjects. This is a cross-sectional study that determined the bacterial level only once, and more samples are needed to identify factors determining the proportion of bifidobacteria. We found an association between antimicrobial treatment of mothers at delivery and bifidobacterial colonization in their infants, but the number of bifidobacteria was not necessarily low in infants with mothers who received antimicrobial treatment at delivery. The reason for this finding is unclear, despite investigation of associations with other factors. The study was conducted as a pilot study to initiate long-term evaluation of the gut microbiota of families, including infants and their mothers. Further follow-up studies with more samples are planned in other regions and facilities to confirm the findings in this study.

In conclusion, an analysis of the gut microbiota in Japanese infants aged 1–2 months old using next-generation sequencing was performed to identify maternal and infant factors that influence the proportion of bifidobacteria in infants. The results indicated that maternal antimicrobial use at delivery has a stronger effect than delivery mode on the gut microbiota, and especially on colonization of bifidobacteria, which dominates the gut in healthy infants. These results provide a better understanding of the gut microbiota in early infants, and suggest the need for follow-up studies from birth in more subjects and in other regions.

### Data deposition

Data from this study are deposited in Figshare. DOI: 10.6084/m9.figshare.5918485.

## Electronic supplementary material

Supplemental Information
