# Supplementary material for: Maternal antimicrobial use at delivery has a stronger impact than mode of delivery on bifidobacterial colonization in infants: a pilot study
Source: J Perinatol. 2018 Jul 24;38(9):1174–81. doi: 10.1038/s41372-018-0172-1 (PMC6128817; doi:10.1038/s41372-018-0172-1)
Supplement: Supplementary file 1 — Supplemental Information [file 41372_2018_172_MOESM1_ESM.docx]

Supplemental information. Relationship of relative abundance of bifidobacteria with background factors

| Factors (n) | | | P value | r_s_ |
| --- | --- | --- | --- | --- |
| Infants with older siblings* | Yes (17) | No (16) | **0.045** |  |
| Exclusively breastfed* | Yes (20) | No (13) | 0.13 |  |
| Sex* | Male (19) | Female (14) | 0.38 |  |
| Mothers with history of allergy* | Yes (16) | No (17) | 0.36 |  |
| Gestational age at birth^†^ | | | 0.98 | 0.37 |
| Birth weight^†^ | | | 0.91 | 0.24 |
| Age of infants when feces was collected^†^ | | | 0.09 | -0.24 |
| Age of mothers^†^ | | | 0.85 | 0.19 |

Significant P values are shown in bold. *Comparison of relative abundance of bifidobacteria for each factor was tested by Mann-Whitney U test. ^†^Correlation between these factors and relative abundance was examined by Spearman rank correlation coefficient (r_s_).
